# Effect of low-moderate intensity traditional Chinese exercises combined with acupuncture on patients with stable chronic obstructive pulmonary disease: study protocol for a randomized controlled trial

**DOI:** 10.3389/fmed.2025.1470196

**Published:** 2025-04-02

**Authors:** Hongxia Duan, Yidie Bao, Linhong Jiang, Peijun Li, Yingqi Wang, Yuchen He, Xinliao Deng, Weibing Wu, Wei Zhang, Xiaodan Liu

**Affiliations:** ^1^School of Rehabilitation Science, Shanghai University of Traditional Chinese Medicine, Shanghai, China; ^2^Department of Rehabilitation, Shuguang Hospital Affiliated to Shanghai University of Traditional Chinese Medicine, Shanghai, China; ^3^Engineering Research Center of Traditional Chinese Medicine Intelligent Rehabilitation, Shanghai University of Traditional Chinese Medicine, Shanghai, China; ^4^Department of Sports Medicine, Shanghai University of Sport, Shanghai, China; ^5^Department of Pulmonary Diseases, Shuguang Hospital Affiliated to Shanghai University of Traditional Chinese Medicine, Shanghai, China

**Keywords:** acupuncture, pulmonary-based Qigong exercise, pulmonary rehabilitation, chronic obstructive pulmonary disease, study protocol

## Abstract

**Background:**

Traditional Chinese exercises (TCEs), as a new technology for pulmonary rehabilitation, have been proven to be effective in patients with chronic obstructive pulmonary disease (COPD). However, further aggravation of dynamic hyperinflation manifested as exertional dyspnea during exercises may limit the partial therapeutic efficacy of TCEs on patients with COPD. Acupuncture therapy, internationally recognized as a complementary and alternative therapy, can effectively improve the degree of dyspnea, and it is expected to serve as an adjuvant therapy for exercise training in patients with COPD to fully realize the therapeutic efficacy of exercise training. Therefore, this study aims to explore the multidimensional and multi-system effects of the combination of pulmonary-based Qigong (PQ) exercise and acupuncture therapy on patients with COPD.

**Methods:**

This protocol describes an assessor-blinded, data analyst-blinded, four-arm randomized controlled trial that aims to recruit 132 participants with stable COPD and randomly allocate them into pulmonary-based Qigong exercise group, acupuncture group, pulmonary-based Qigong exercise and acupuncture combined group, or control group at a 1:1:1:1 ratio. All participants will receive usual medical care and health education; those in the intervention groups will receive PQ exercise, acupuncture treatment, or a combination of both treatments three times per week for 8 weeks. The primary outcome will be the exercise endurance as assessed by a 6-min walk test. Secondary outcomes will include lung function, degree of dyspnea, diaphragmatic function, respiratory muscle strength, skeletal muscle structure, skeletal muscle function, psychological states, and quality of life. Exploratory outcomes will include the levels of inflammatory mediators. The frequency and severity of acute exacerbations of COPD will be recorded at baseline and 1 year after intervention.

**Discussion:**

The findings of this study will clarify the effects of the combination of PQ exercise and acupuncture therapy on the multi-system function of patients with stable COPD to provide evidence for acupuncture as an adjuvant therapy for pulmonary rehabilitation.

**Clinical trial registration:**

https://www.chictr.org.cn, ChiCTR2300076255

## Introduction

Chronic obstructive pulmonary disease (COPD), a common chronic respiratory disease, is characterized by chronic respiratory symptoms (dyspnea, cough, and sputum) caused by airway lesions and/or abnormal alveoli, and it causes progressive and not fully reversible airflow limitation (1). COPD is one of the top three causes of death worldwide, with a prevalence rate of 13.7% among people aged 40 years and above in China (2). Its prevention and treatment are major public health challenges, and the global economic burden of COPD is expected to continue to increase in the coming decades, especially in China and the United States, due to continued exposure to pathogenic risk factors and aging population (3, 4). Therefore, the prevention and treatment situation of COPD is severe and deserves attention globally.

COPD is a complex multi-system disease in older adult people with multiple comorbidities (5). It not only remarkably affects lung function but also leads to considerable extra-pulmonary adverse reactions, including decreased motor function, impaired skeletal muscle function, and diaphragmatic dysfunction, thus seriously affecting the psychological states and quality of life (QoL) of patients (6–10). Studies have demonstrated a significant reduction in the 6-min walking distance (6MWD) of patients with COPD compared with age-matched healthy individuals (11). Furthermore, skeletal muscle dysfunction is prevalent in over one-third of patients with COPD (12), characterized by diminished muscle strength, endurance, and explosiveness (13). It is closely associated with exacerbations and increased hospitalizations (10). Skeletal muscle dysfunction significantly affects posture and balance control abilities while increasing the risk of falls (14, 15). Meanwhile, diaphragmatic dysfunction is observed throughout all stages of COPD progression (16). Zhou et al. (17) identified diaphragmatic atrophy and dysmotility in patients with COPD, indicating an inability to meet prolonged demands on key respiratory muscles. Such inability further exacerbates dyspnea symptoms and muscle fatigue (18). Consequently, comprehensive treatment strategies targeting multiple dimensions and systems are imperative for patients with COPD.

Exercise-centered pulmonary rehabilitation (PR) program is the most efficacious treatment strategy for enhancing respiratory dysfunction and exercise endurance in patients with COPD, as pointed out in the Global Initiative for COPD 2024 Report (3). As a new PR technique, traditional Chinese exercises (TCEs) have obvious advantages of safety, efficiency, and economy. Their efficacy in COPD rehabilitation has been confirmed by an increasing number of randomized controlled trials (19–22). Moreover, TCEs, such as pulmonary-based Qigong (PQ), Taichi, Liuzi jue, and Baduanjin, have been accepted by an increasing number of patients in clinical practice (23). Gilliam et al. (24) found that twice-weekly Taichi improved fatigue, exercise endurance, and dyspnea and provided positive effects on psychological states and self-management awareness. Multiple systematic reviews and meta-analyses have shown that Baduanjin can improve lung function, exercise endurance, psychological states, and QoL in patients with stable COPD (25–27). A set of specific exercise program for COPD, namely, PQ, was created on the basis of Wuqinxi, Liuzijue, Baduanjin, and Yijinjing Qigong to further improve the intervention effect of TCEs on patients with COPD. This program can effectively improve the exercise endurance, anxiety, depressive mood, and QoL of patients with stable COPD (19). However, the destruction of lung parenchyma and the airway abnormalities caused by chronic inflammation lead to the obstruction of exhaled airflow (28), which increases the demand for ventilation during exercise in patients with COPD, resulting in a forced decrease in exhalation time and further aggravating dynamic hyperinflation (29, 30). This phenomenon partially limits the efficacy of exercise rehabilitation. How to reduce the dynamic hyperinflation and improve the tolerance during exercise have become the key issues to fully realize the efficacy of PR.

Acupuncture, an internationally recognized complementary and alternative therapy, is anticipated to be utilized as an adjuvant therapy alongside exercise training in patients with COPD, aiming to optimize the benefits of exercise training by improving dynamic hyperinflation. In recent years, acupuncture has demonstrated enhancement in bronchiectasis function (31), alleviation of dyspnea symptoms (32, 33), decreased risk of acute exacerbation, and improved diaphragm and peripheral skeletal muscle performance to a certain extent while enhancing exercise endurance (34–36). These effects may be attributed to the anti-inflammatory effects of acupuncture (31, 37). Jiang et al. (37) summarized the current animal studies on the application of acupuncture in COPD and found that different acupuncture types and acupoints have similar effects on modulating the levels of inflammatory cytokines. Acupuncture exerts anti-inflammatory effects by inhibiting the release of inflammatory cells, inflammasomes, and inflammatory cytokines, and the main underlying mechanism through which acupuncture improves inflammation in COPD is the modulation of relevant signaling pathways such as nuclear factor (NF)-κB, mitogen-activated protein kinase signaling pathways, cholinergic anti-inflammatory pathway, and dopamine D2 receptor pathway (37). However, studies on the efficacy of TCEs combined with acupuncture in the treatment of patients with COPD are relatively few, and the existing studies have certain limitations. The efficacy indicators are not sufficiently comprehensive, and most of them are subjective scales, lacking a multidimensional and multi-system evaluation system combining subjective and objective evaluation methods.

Therefore, this study will comprehensively evaluate the efficacy of the combination of PQ exercise and acupuncture on exercise endurance, lung function, dyspnea, diaphragmatic function, respiratory muscle strength, skeletal muscle structure, skeletal muscle function, psychological states, QoL, and frequency and severity of acute exacerbation in patients with stable COPD. It will also explore the potential mechanism from the perspective of anti-inflammatory effects. All three intervention regimens are hypothesized to be significantly positive, and that the combination therapy has optimal benefits in patients with COPD compared with single intervention.

## Methods and analysis

Patient and public involvement (No patient involved).

### Study design

**Figure 1:**
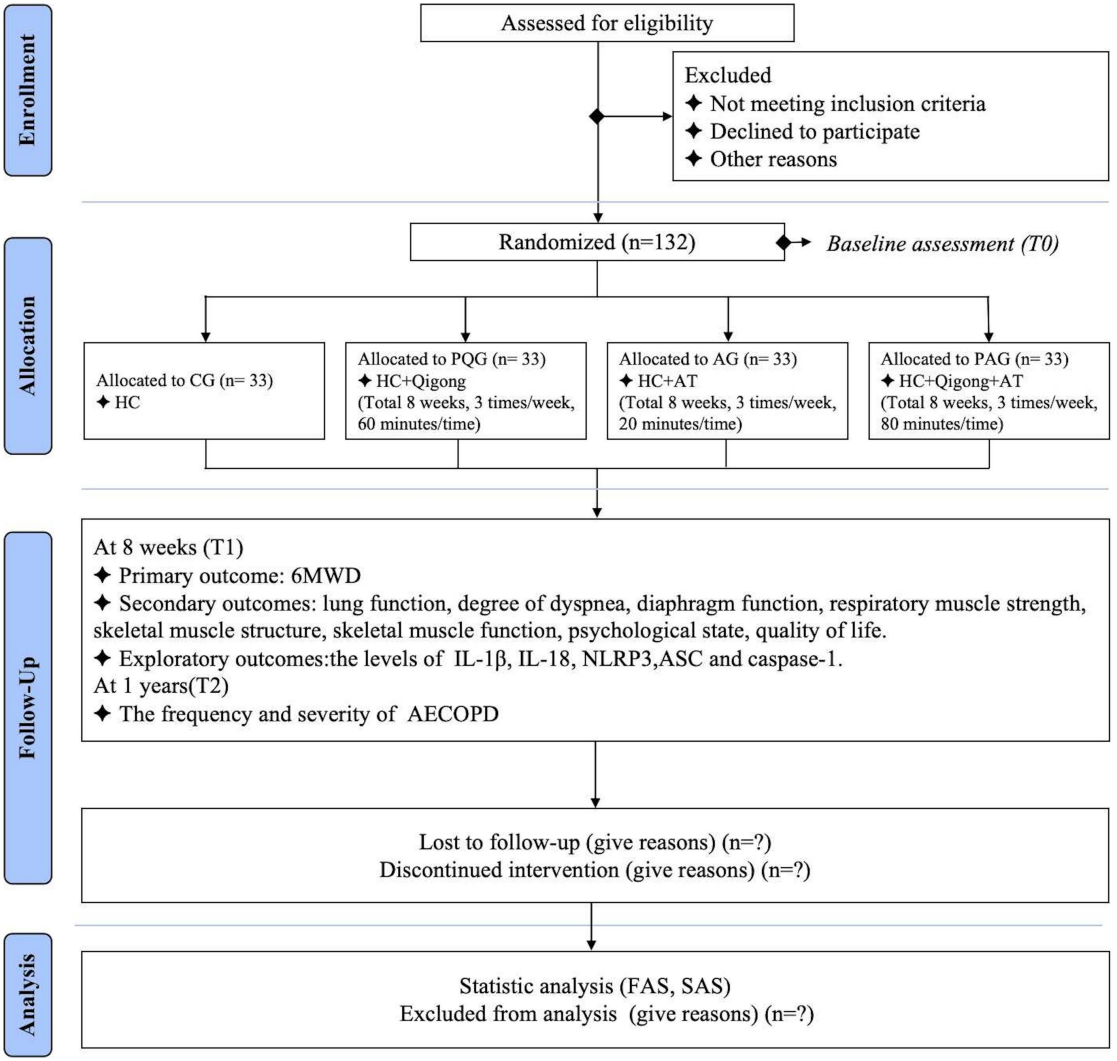
Flow diagram of the study. The template is from the CONSORT 2010 flow diagram. CG, control group; PQG, pulmonary-based Qigong group; AG, acupuncture group; PAG, pulmonary-based Qigong exercise and acupuncture combined group; HC, health education and conventional medical treatment; AT, acupuncture treatment; 6MWD, 6-min walking distance; IL, interleukin; NLRP3, NOD-like receptor protein 3; ASC, apoptosis-associated speck-like protein containing a C-terminal caspase recruitment domain; caspase-1, cysteinyl aspartate-specific proteinase-1; AECOPD, acute exacerbation of chronic obstructive pulmonary disease; FAS, full analysis set; SAS, safety assessment set.

This study is a single-center, assessor-blinded, data analyst-blinded, four-arm randomized controlled trial to be conducted at Shuguang Hospital Affiliated to Shanghai University of Traditional Chinese Medicine (TCM) in accordance with the Consolidated Standards of Reporting Trials (CONSORT) 2010 statement and the Standards for Reporting Interventions in Clinical Trials of Acupuncture (STRICTA) criteria (38, 39). A total of 132 participants will be recruited between October 2023 and October 2024, and randomly allocated to PQ exercise group (PQG), acupuncture group (AG), PQ exercise and acupuncture combined group (PAG), or control group (CG) at a 1:1:1:1 ratio. All participants will receive usual medical care and health education; those in the three intervention groups will receive PQ exercise, acupuncture treatment, or a combination of both treatments three times per week for 8 weeks. The primary outcome will be exercise endurance as assessed by a 6-min walk test (6MWT). Secondary outcomes will include lung function, degree of dyspnea, diaphragmatic function, respiratory muscle strength, skeletal muscle structure, skeletal muscle function, psychological states, and QoL. Exploratory outcomes will include the levels of inflammatory mediators. Outcomes will be measured within 1 week before and after the interventions. In addition, the frequency and severity of acute exacerbation of COPD (AECOPD) will be recorded at baseline and 1 year after intervention. A flow diagram of the study design is shown in Figure 1, and the study schedule is depicted in Table 1. This study has received ethics approval from Shuguang Hospital Affiliated to Shanghai University of TCM (approval number 2023–1360–127-01), and it has been registered in the Chinese Clinical Trial Registry (ChiCTR2300076255). The study protocol strictly follows the Standard Protocol Items: Recommendations for Interventional Trials (SPIRIT) checklist (Supplementary File 1).

**Table 1:** Schedule of enrolment, intervention and assessment.

Time point	Enrollment	Allocation	Treatment phase		Follow-up
	Week-1	Week0	Week1	Week8	Week60
Assessment of eligibility:					
Eligible screen	X				
Informed consent	X				
Randomization		X			
Allocation		X			
Intervention:					
CG				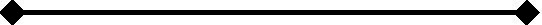	
PQG			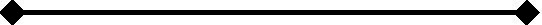		
AG			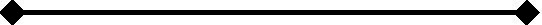		
PAG			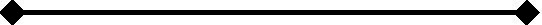		
Primary outcome:					
6MWD		X		X	
Secondary outcomes:					
Lung function		X		X	
mMRC		X		X	
Diaphragmatic function		X		X	
MIP & MEP		X		X	
Skeletal muscle structure		X		X	
Skeletal muscle function		X		X	
HAMA&HAMD		X		X	
SGRQ		X		X	
AECOPD		X		X	X
Exploratory outcomes:					
IL-1β, IL-18, NLRP3, ASC, caspase-1		X		X	

CG, control group; PQG, pulmonary-based Qigong group; AG, acupuncture group; PAG, pulmonary-based Qigong exercise and acupuncture combined group; 6MWD, 6-min walking distance; mMRC, Modified Medical Research Council; MIP, maximal inspiratory pressure; MEP, maximal expiratory pressure; HAMD, Hamilton Anxiety Scale; HAMD, Hamilton Depression Scale; SGRQ, St. George’s Respiratory Questionnaire; AECOPD, acute exacerbation of chronic obstructive pulmonary disease; IL, interleukin; NLRP3, NOD-like receptor protein 3; ASC, apoptosis-associated speck-like protein containing a C-terminal caspase recruitment domain; caspase-1, cysteinyl aspartate-specific proteinase-1.

### Sample size calculation

This study is a superiority trial. The sample size was calculated on the basis of 6MWD by using PASS software (version 15.0). Based on the previous clinical studies of TCEs and acupuncture intervention for COPD (19, 40, 41), the added values of 6MWD after the intervention of TCEs, acupuncture, and exercise-combined therapy were estimated to be 34.5, 32, and 38, respectively, with the assumption that the mean value of 6MWD in the control group was 297.9 and the standard deviation was 37.4. By setting alpha to 0.0083 (two-sided) and the power to 0.80, a minimum sample size of 26 participants per group was calculated. Considering a 20% attrition rate, a total of 132 participants with 33 participants per group is a reasonable sample size for this study.

### Participants

Participants with confirmed COPD (42), on stable treatment for at least for 4 weeks before randomization (confirmed by absence of acute exacerbation and related drug changes), between the ages of 40 and 80 years, and who are capable and willing to provide consent are eligible to participate. The exclusion criteria are as follows: (1) coexistence of other respiratory diseases, including asthma, tuberculosis, bronchiectasis, and lung cancer; (2) severe medical condition, such as in the heart, brain, blood vessels, liver, kidneys, endocrine system, nervous system, or musculoskeletal system, that may affect exercise capacity; (3) coexistence of open injuries that are not suitable for muscle strength assessment and acupuncture treatment; (4) presence of severe speech, attention, hearing, visual, intellectual, psychiatric, or cognitive impairment that makes the individual unable to follow instructions properly; (5) women of childbearing age who were pregnant or lactating, planned to have children within 6 months, and could not take effective contraceptive measures during the trial period; (6) conditions that are not suitable for acupuncture treatment, such as dizziness, skin damage, infection, and ulcer at the acupuncture site; (7) participation in regular exercise training (at least two times per week, 60 min each time) in the past 6 months; (8) received acupuncture treatment 1 month before the trial. We will recruit potential subjects through a variety of ways, such as following up in the respiratory department of Shuguang Hospital, poster recruitment in the community service center, publicity on WeChat, and publicity in the surrounding community to reach target sample size.

### Randomization

The randomization process will be conducted by an independent investigator. First, a total of 132 random numbers ranging from 1 to 1,000 will be generated and assigned randomly to each subject by IBM SPSS (version 24.0). These numbers will then be arranged in ascending order. Specifically, subjects in the order of 1–33, 34–66, 67–99, and 100–132 will be allocated to PQG, AG, PAG, and CG, respectively.

### Blinding

Blinding of subjects and interveners is not feasible due to the inclusion of exercise and acupuncture interventions in this study. However, assessors will remain blinded. All participants and assessors will receive strict instructions not to discuss or disclose any information regarding the intervention methods prior to the start of assessments. Independent research personnel will be present at all times to ensure successful blinding procedures are maintained consistently throughout data collection processes. In cases where assessors become aware of a subject’s intervention method during assessment, their involvement will be terminated promptly with replacement by another assessor.

### Interventions

#### Control group

CG will receive usual medical care in accordance with the international guidelines (42, 43), and health education every 2 weeks, covering the concept, risk factors, and self-management of COPD. During the trial, the patients will be asked to maintain their extant daily habits, and regular weekly telephone calls will be conducted to ask them about their medications, exercise, and daily activity for the week to ensure adherence to the trial.

#### PQ exercise group

**Figure 2:**
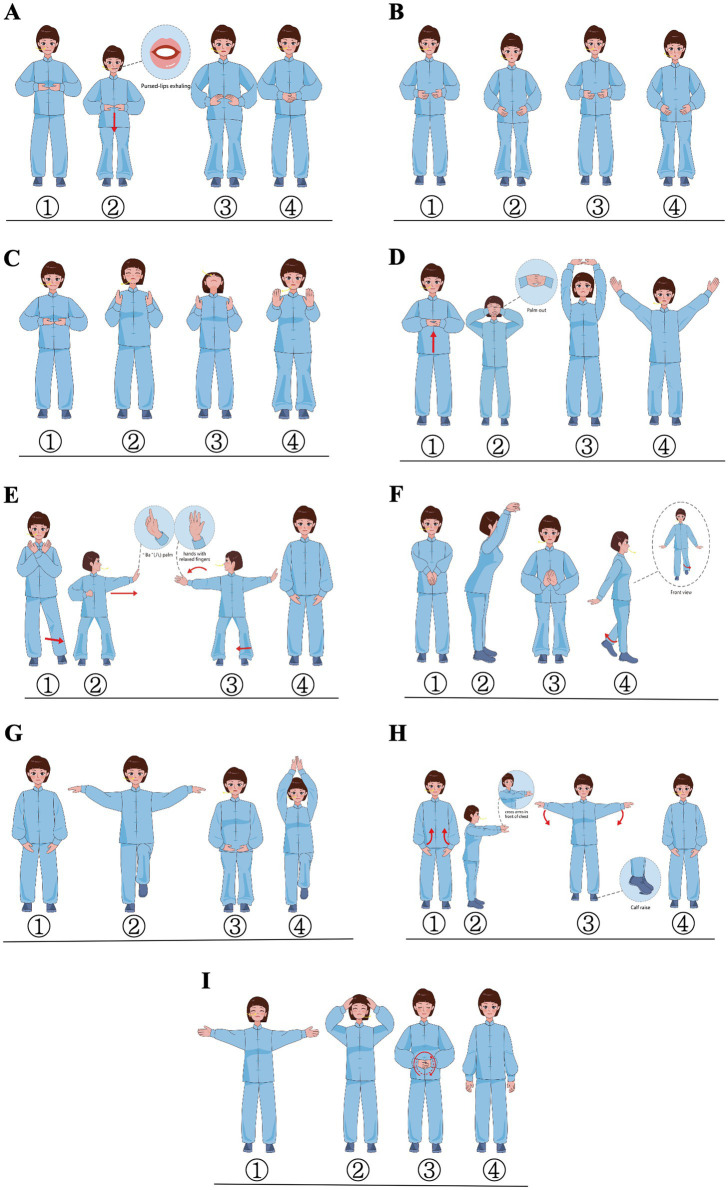
Demonstration of pulmonary-based Qigong exercise. The figure is a schematic diagram of nine actions, mainly including movements of the body and limbs, rhythm of exhalation and inhalation, and the direction of eye observation. **(A)** Rise-up position; **(B)** “Hu” sounding; **(C)** “Si” sounding; **(D)** Pushing up the sky to regulate the triple warmer; **(E)** Drawing a bow to shoot a vulture; **(F)** Crane extension in the crane exercise; **(G)** Crane fly in the crane exercise; **(H)** Cross-armed iron staff; **(I)** Restore position.

PQG will receive an 8-week intervention of PQ exercise (44), three times per week for 60 min per session (Figure 2). Two weeks before the intervention, a coach with more than 20 years of Qigong experience will train and assess the three researchers who are responsible for teaching the participants to ensure that these researchers master all the points of Qigong. During the intervention period, exercise groups consisting of 6–12 patients will be formed, and each group will receive step-by-step instructions from the three researchers. The training sessions will comprise three 1-h sessions per week, totaling 24 sessions, of which the training structure includes warm-up, core activities, and cooldown. In 1–6 training sessions, the patients will be systematically taught to master all the points of Qigong. In 7–24 training sessions, the patients will practice PQ exercises together with the researchers. Further details regarding the intervention protocol can be found in Supplementary File 2. The attendance required is no less than 85%. The training load will be monitored using the Borg CR10 scale at a level of 4–6 (42). If a subject cannot perform a specific exercise at the right intensity, he or she could perform at best achieved intensity, and the researchers will record the performance. If the subjects feel tired during the training, they could take a break and continue to complete the training. The exercise place is an independent and spacious place with suitable temperature and good ventilation. It could accommodate more than 20 people, and it is equipped with effective first-aid facilities.

#### Acupuncture group

**Figure 3:**
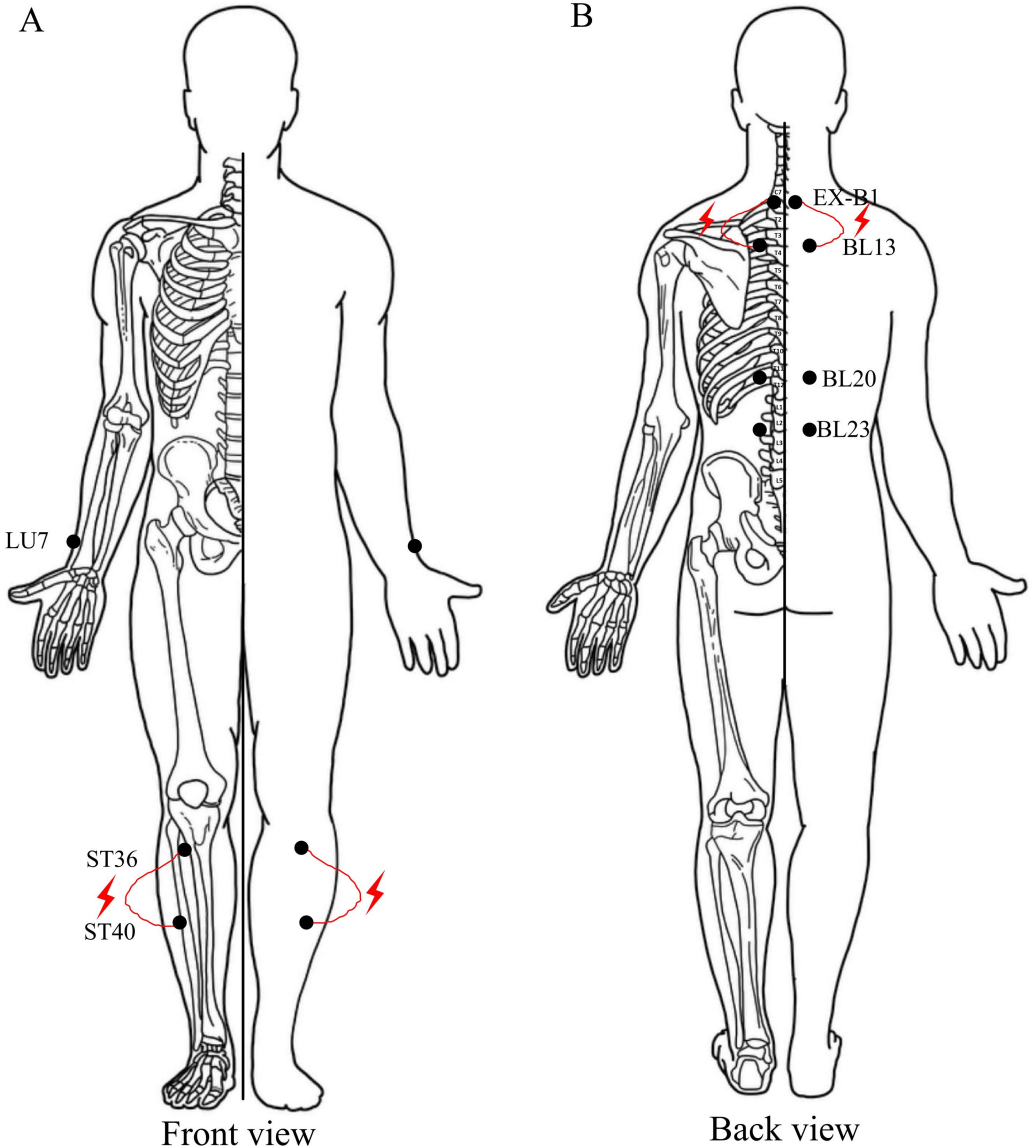
Details of acupuncture therapy. The red mark indicates the acupoint and connection mode of electroacupuncture.

AG will receive an 8-week intervention of acupuncture therapy, three times per week, for 20 min per session (Figure 3). The selected acupoints are Lieque (LU7), Dingchuan (EX-B1), Feishu (BL13), Pishu (BL20), Shenshu (BL23), Zusanli (ST36), and Fenglong (ST40). The acupoints are selected bilaterally. The location of acupoints and the direction and depth of needle insertion are shown in Table 2 (45, 46). Treatment will be performed by a licensed acupuncturists who have at least 3 years of experience with acupuncture. The disposable sterile acupuncture needles of Huatuo brand are selected, with specifications of 0.3 × 40 mm and 0.3 × 25 mm. The acupuncturist will instruct the patient to assume a seated position and proceed to sterilize the targeted area by using sterile tweezers and a cotton ball soaked in 75% alcohol, followed by performing acupuncture techniques to ensure the attainment of “*de qi*” sensation (a composite of sensations including soreness, numbness, distention, heaviness, and other sensations), which is considered an important element in the efficacy of acupuncture (47). Then, an electroacupuncture device (SDZ-III, Huatuo, China) with a frequency of 2 Hz and continuous wave will be attached to the needle handles at the bilateral BL20 and EX-B1, ST36 and ST40, respectively, forming four circuits. The patients will be instructed to refrain from drinking alcohol, strong tea, coffee, high-intensity exercise, and over-hunger and to remain calm before treatment. The acupuncture site is far away from the exercise site, and it is an independent place with suitable temperature and safe environment, equipped with effective first-aid facilities.

**Table 2:** Location of acupoints and direction and depth of needle insertion.

Acupoint	Area	Location	Needle insertion
Lieque (LU7)	Lateral forearm	In the depression of the abductor pollicis longus tendon groove between the extensor pollicis brevis tendon and abductor pollicis longus tendon, which is 1.5 cun above the transverse line of the distal carpal volar.	Angled upward, 0.3–0.5 cun.
Dingchuan (EX-B1)	Cervical region	0.5 cun horizontally next to the depression below the spinous process of the 7th cervical spine.	Vertically or slightly inward, 0.5–1 cun.
Feishu (BL13)	Dorsal region	1.5 cun horizontally next to the depression below the spinous process of the 3rd thoracic vertebra.	Lateral inclination of 45 degrees, 0.5–1 cun.
Pishu(BL20)	Dorsal region	1.5 cun horizontally next to the depression below the spinous process of the 11th thoracic vertebra.	Vertically, 0.5–1 cun.
Shenshu (BL23)	Lumbar region	1.5 cun horizontally next to the depression below the spinous process of the 2nd lumbar spine.	Vertically, 0.5–1 cun.
Zusanli (ST36)	Lateral calf region	3 cun below the Dubi (ST35) on the line between Dubi (ST35) and Jiexi (ST41). Note: ST35 is located in the depression on the lateral side of the patellar ligament in the anterior region of the knee; ST41 is located in the depression in front of the ankle joint between the extensor pollicis longus tendon and the extensor digitorum longus tendon.	Vertically, 1–2 cun.
Fenglong (ST40)	Lateral calf region	8 cun above the tip of the lateral malleolus, lateral margin of the tibialis anterior muscle, 1 cun outside the Tiaokou (ST38). Note: ST38 is 8 cun below ST35 on the lateral side of the calf and on the line between ST35 and ST41.	Vertically, 1–1.5 cun.

Cun is the unit of length. In this paper, the width of the interphalangeal joint of the patient’s thumb is 1 cun. When the second to fifth fingers of the patient are closed, the width of the four fingers is 3 cun based on the transverse lines of the proximal interphalangeal joint of the middle finger.

#### PQ exercise and acupuncture combined group

PAG will receive an 8-week intervention of PQ exercise combined with acupuncture therapy three times per week for 80 min per session. The time between the two treatments will be at least 1 h to ensure that the patients are well rested and in good condition.

### Outcome measures

#### Primary outcome

The primary objective of this study is to evaluate the change in exercise endurance after an 8-week intervention. Exercise endurance will be assessed by 6MWT. In accordance with the American Thoracic Society guidelines for 6MWT, a closed, long, straight, flat corridor in the room will be selected; a 30-meter scale will be placed on the ground; and the starting points of both ends will be marked with bright color bands. The patients will be asked to walk back and forth as fast as they could within 6 min, and the maximum distance walked will be recorded as 6MWD (48).

#### Secondary outcomes

- Lung function will be assessed using a spirometer (MasterScreen-Body/Diff, CareFusion Germany 234 GmbH, Germany) by a professional technician in accordance with the American Thoracic Society/European Respiratory Society guidelines (49). The outcomes include forced vital capacity, forced expiratory volume in 1 s/forced vital capacity (FEV1/FVC), predicted FEV1% (FEV1%pred), forced expiratory flow at 25% of forced vital capacity predicted (FEF25%), FEF50%, FEF75%, and maximum mid-expiratory flow after bronchodilator inhalation.
- The degree of dyspnea in patients will be measured using the modified Medical Research Council Dyspnea Scale, which is a 5-point scale (0–4) on which higher scores indicate worse levels of dyspnea (42).
- Diaphragmatic function will be evaluated by a color Doppler ultrasonic diagnostic apparatus (Aplio i900TUS-AI900, Canon, Japan) with a linear array probe (10–18 MHZ, i18LX5) in M-mode. Two experienced and trained sonographers will perform the procedure. First, the sonographer will instruct the patients to assume a seated position and place the probe at the junction of the right anterior axillary line and the 7th–9th intercostal space, ensuring that the sampling line is perpendicular to the diaphragm (50). Subsequently, precise adjustments will be made for each individual to optimize visualization of dynamic diaphragmatic images. Finally, clear and stable recordings of dynamic diaphragmatic images under resting breathing will be obtained. The diaphragm thickness at end-expiration and end-inspiration (the distance between the parallel hyperechoic medial sides of the diaphragm) and the contraction velocity of the diaphragm (displacement/time) will be measured and recorded by the sonographer. The measurements will be repeated three times, and the average value will be taken. Besides, the diaphragm thickening ratio ((mean thickness at end-inspiration – mean thickness at end-expiration)/mean thickness at end-expiration) will be calculated and recorded.
- The maximal inspiratory pressure (MIP) and maximal expiratory pressure (MEP) of respiratory muscle strength will be measured using a respiratory muscle strength tester (TA1, BreathHome, China). The patients will be asked to use a mouthpiece to ensure no air leakage in the mouth and nose. After breathing stably, the patients will be asked to inhale hard and quickly for more than 3 s, and the MIP will be automatically calculated by the software. After breathing stably, the patients will exhale hard and quickly for more than 3 s, and the MEP will be automatically calculated by the software. The measurements will be repeated three times with a 1-min rest interval to take the best value.
- Skeletal muscle structure will be measured by a color Doppler ultrasonic diagnostic apparatus (Aplio i900TUS-AI900, Canon, Japan) with a linear array probe (10–18 MHZ, i18LX5) in M-mode. First, The patients will be asked to lie in a supine position, breathe calmly, with their upper limbs extended and palms up and naturally placed at the side of the body. The probe will be placed at the middle and upper 1/3–1/2 of the starting and end points of the biceps brachii muscle to collect clear and stable images. The thickness of the soft tissue in front of the humerus and the cross-sectional area of the biceps brachii muscle will be calculated and recorded. Second, the probe will be placed at 1/2–3/5 of the distance from the anterior superior iliac spine to the upper edge of the patella to collect clear and stable images of the patient in supine position while the lower limb is extended. The thickness of the soft tissue in front of the femur and the cross-sectional area of the rectus femoris muscle will be calculated and recorded. All the above tests will be repeated three times, and the average value will be taken.
- Skeletal muscle function will be assessed using an isokinetic muscle strength testing system (HUMAC NORM, CSMi, American) and a wireless bio-signal acquisition system (Delsys Inc., Boston, United States) to evaluate the strength and endurance of the major skeletal muscles of the upper and lower limbs. Isokinetic muscle strength tests will be performed at slow (60°/s) and fast (180°/s) angular velocities on the elbow and knee joints separately by a technician in accordance with the manual of the instrument. At an angular velocity of 60°/second, the patients will be instructed to perform five consecutive flexion and extension exercises with their maximum effort within the designated range of motion, and peak torque will be recorded. At an angular velocity of 180°/second, the patients will be asked to complete 30 consecutive flexion and extension exercises with their maximum effort throughout the entire range of motion, and total work and fatigue index are recorded. Moreover, surface electromyography signals from two 6-s maximal static contraction tests and one exhaustive static contraction test will be collected for analysis to measure the root mean square, median frequency, and mean power frequency.
- The psychological states of the participants will be evaluated using the Hamilton Anxiety Scale (HAMA) and the Hamilton Depression Scale (HAMD), which will be completed through self-assessment (51). The HAMA, consisting of 24 items scored from 0 to 4, will assess anxiety symptoms. The HAMD, consisting of 24 items rated on a scale of 0–2 or 0–4, will evaluate depressive symptoms. Higher scores indicate higher levels of anxiety and depression.
- QoL will be assessed utilizing the St. George’s Respiratory Questionnaire, a specific assessment tool for patients with COPD (52). This questionnaire comprises a total of 50 questions that cover three main aspects: symptoms, activities, and effect on daily activities. Each question is scored on a scale ranging from 0 to 100 on the basis of its significance, with lower scores indicating no impairment and higher scores indicating extreme impairment.
- The frequency and severity of AECOPD 1 year before and after the intervention will be recorded.

#### Exploratory outcomes

A 10 mL venous blood sample will be collected from the patients under fasting conditions before and after the 8-week intervention. Plasma and serum samples will be stored at −80°C as required. The levels of inflammatory cytokines interleukin (IL)-1β and IL-18 will be tested using ELISA kits. The content of NOD-like receptor protein 3 (NLRP3), apoptosis-associated speck-like protein containing a C-terminal caspase recruitment domain (ASC), and cysteinyl aspartate-specific proteinase (caspase)-1 will be measured using polymerase chain reaction.

### Quality control

The Physical Activity Scale for the older adult will be used for monitoring the daily activities of all participants during the intervention period (53). Four WeChat groups will be established for the four groups to answer all participants’ questions about the disease, organize collective health education lectures intermittently, send some gifts, and maintain effective contact with them to ensure high compliance and an attendance rate of more than 85%. During the intervention period, one-on-one theoretical and practical teaching, including movements, breathing, and mind, will be provided to help the participants master all the key points of the PQ and pass the assessment conducted by a coach with 10 years of exercise experience to ensure the fidelity of the exercise training. Meanwhile, the participants will be asked to come to Shuguang Hospital for intensive exercise at least three times per week, and for other home exercises, training videos will be asked to be sent to the recorder.

To control for potential bias due to dietary factors, participants’ daily dietary patterns and the intake of nutritional supplements will be recorded using food diaries throughout the intervention period (54). These records will provide detailed information on daily food intake and supplement use, and will be analyzed together with other health behavior data to ensure that the intervention’s effects are not confounded by diet-related factors.

### Data management

The clinical trial data management database EpiData3.1 software will be used to ensure the accuracy of all data through dual person and dual recording. The database can only be accessed utilizing a unique password that only a specific researcher knows.

### Statistical analysis

An independent statistician who is not involved in outcome measurement will perform statistical analysis using SPSS software (version 24.0). All participants will be included in the intention-to-treat analyses. The multiple imputation method will be utilized to analyze missing data. Continuous data will be expressed as mean ± SD or median ± quartile interval, and enumeration data will be expressed as a relative ratio. Comparisons of baseline differences between groups will be performed using ANOVA or the Kruskal–Wallis H test for continuous variables and χ2 for categorical variables. Comparisons within each group (before and after 8 weeks of intervention) will be evaluated using a paired t-test or non-parametric test. Comparisons of endpoint differences between groups will be assessed using a 2 × 2 ANOVA (group × time) or the Kruskal–Wallis H test, with analysis of covariance applied when baseline is not uniform. In addition, a Difference-in-Difference (DID) analysis will be conducted to further assess the intervention effects while accounting for baseline differences and time trends.Multiple comparisons between the intervention arms will be performed using SNK-q test or the Nemenyi test. A *p* value of <0.05 will be considered statistically significant.

To further evaluate the discriminatory ability of each intervention in improving exercise tolerance, the area under the receiver operating characteristic curve (AUC) will be calculated for each intervention group, using the improvement in 6MWD as the classification criterion. Treatment effectiveness will be defined as an improvement in 6MWD ≥ 54 meters (minimal clinically important difference) (55). AUCs will be compared using the DeLong test with MedCalc software (MedCalc Software Ltd., Ostend, Belgium). A *p* value of <0.05 will be considered statistically significant.

### Adverse events

All adverse events will be recorded in the case report form throughout the trial. Adverse events related to exercise and acupuncture (such as muscle and joint injuries, severe pain, local hematoma, infection, and abscess), including discomfort after treatment, will be promptly and in detail recorded. The assessor will collect from the patient a detailed description of the category and severity of the adverse event, and any correlation with the treatment. If an adverse event is serious and related to the trial, the patient will be withdrawn from the study and receive appropriate medical care.

### Dissemination plans

The results of this study will be fully disclosed in an international peer-reviewed journal, and all positive and negative results will be reported.

## Discussion

This paper presents a clinical study protocol aiming to investigate the effect of the combination of PQ exercise and acupuncture therapy on multisystem function in stable patients with COPD, and the results will provide effective evidence for acupuncture as an adjuvant therapy for PR.

The acupuncture points for COPD treatment were determined on the basis of acupoint selection rules found in current literature and with guidance from experts in the field. Shi et al. (31) discovered that in clinical trials, the chosen acupoints are primarily located on the back, waist, chest, and abdomen. High-frequency acupoints include Dingchuan (EX-B1), Feishu (BL13), Zusanli (ST36), and other relevant acupoints include Pishu (BL20) andShenshu (BL23). Liu Y et al. analyzed, through data mining technology, that BL13, ST36, EX-B1, and BL23 are high-frequency acupoints (56). According to the Clinical Application Guide of TCM PR for COPD, patients with stable COPD should receive acupuncture at the main acupoints, such as BL13, EX-B1, and Lieque (LU7), whereas those with excessive phlegm should be treated at ST36, Zhongwan (RN12), Fenglong (ST40), and Danzhong (RN17). The needles should be retained for a duration of 15–30 min once every 2 or 3 days over a course of 2 months (57). On the basis of the above information; under the guidance of senior acupuncture doctors in Shuguang Hospital; and considering clinical safety, high efficiency, operability, and compliance, the acupoints LU7, EX-B1, BL13, BL20, BL23, ST36 and ST40 were finally selected in this clinical trial. The intervention time was 20 min/time, three times/week, and 8 weeks. The acupoints in this study is symptomatic of the TCM pathogenesis of COPD, characterized by lung, spleen, and kidney deficiencies; phlegm turbidity; and blood stasis (58). Stimulating BL13, BL20, and BL23 can regulate the lung qi, spleen qi, and kidney qi for treating cough and asthma symptoms while improving respiratory function. ST36 is the he-sea point of Yangming Stomach Meridian of Foot that supplements spleen to nourish the lungs (59). LU7, EX-B1, and ST40 are commonly used acupoints for relieving cough and asthma (59–62). The combination of all these acupoints embodies a holistic approach in treating lung, spleen, and kidney together to tonify lung and kidney functions while strengthening earth (spleen) to generate metal (lung), ultimately facilitating expectoration, suppressing cough, and relieving dyspnea.

The anti-inflammatory effects of acupuncture may play an important role in the treatment of COPD (31, 37). Many animal studies and clinical trials have demonstrated that NLRP3 inflammasome is activated and upregulated in COPD models and patients with COPD compared with those without COPD, and that it is involved in the occurrence and progress of chronic inflammation in COPD (63–66). The NLRP3 inflammasome, consisting of NLRP3, ASC, and caspase-1, plays a crucial role in innate immunity. Upon sensing signal stimulation, the NLRP3 protein acts as a scaffold to bind ASC. Subsequently, ASC recruits and cleaves pro-caspase-1 into its active form, leading to the processing, maturation, and release of inflammatory factors such as IL-1β and IL-18 (67). Zou and Liu et al. (68, 69) discovered that 2 weeks of acupuncture therapy at BL13 and ST36 could downregulate the protein expression levels of NLRP3, caspase-1, and ASC in lung tissues. This finding resulted in reduced levels of IL-1β in bronchoalveolar lavage fluid, alleviated lung inflammation, and improved lung injury in COPD mice. Therefore, the present study proposes that acupuncture could inhibit the activation of the NLRP3 inflammasome while reducing the protein expression levels of caspase-1 and ASC. Additionally, it could decrease the release of IL-1β and IL-18 inflammatory cytokines while mitigating lung tissue inflammation and consequently improving lung function and relieving dyspnea symptoms among patients with COPD.

In this study, 6MWD will be utilized as the primary outcome to objectively assess the functional exercise capacity of patients with COPD, which is a systemic disease with numerous extrapulmonary effects, including cardiovascular disease, frailty, and sarcopenia. Compared with FEV1%, 6MWD comprehensively reflects the intrapulmonary and extrapulmonary effects on cardiopulmonary function, skeletal muscle function, and daily physiological activity in patients with COPD (70), which in small studies has been predictive of morbidity and mortality in in patients with severe COPD (71). 6MWT is a cost-effective measure that does not require expensive equipment or complex technology. It is easy to administer, safe for subjects to perform, and well-tolerated by them. Therefore, 6MWD has been widely adopted as the primary outcome in studies related to COPD PR (72–76).

## Strength

The advantages of this study are as follows: (1) This study aims to establish a comprehensive efficacy evaluation system for the combination of PQ exercise and acupuncture therapy in patients with COPD, encompassing exercise endurance, lung function, degree of dyspnea, diaphragmatic function, respiratory muscle strength, skeletal muscle structure, skeletal muscle function, psychological states, QoL, and frequency and severity of AECOPD. (2) This study will utilize a color Doppler ultrasound diagnostic instrument, a respiratory muscle strength tester, an isokinetic muscle strength testing system, and a wireless biological signal acquisition system to enhance the objectivity and accuracy of the evaluation results. (3) PQ exercise and acupuncture therapy have the characteristics of low cost, high safety, and easy acceptance by patients, thus warranting future promotion.

## Conclusion

Exercise-centered PR programs have been proven to be effective for patients with COPD. However, further aggravation of dynamic hyperinflation manifested as exertional dyspnea during exercises may limit the partial therapeutic efficacy of PR on patients with COPD. Acupuncture therapy, internationally recognized as a complementary and alternative therapy, can effectively improve the degree of dyspnea and is expected to serve as an adjuvant therapy for exercise training in patients with COPD to fully realize therapeutic efficacy of exercise training. Therefore, this study aims to explore the multidimensional and multisystem effects of the combination of PQ exercise-centered PR with acupuncture therapy on patients with COPD.
