# Supplementary material for: Effect of low-moderate intensity traditional Chinese exercises combined with acupuncture on patients with stable chronic obstructive pulmonary disease: study protocol for a randomized controlled trial
Source: Front Med (Lausanne). 2025 Apr 2;12:1470196. doi: 10.3389/fmed.2025.1470196 (PMC12000029; doi:10.3389/fmed.2025.1470196)
Supplement: Supplementary file 2 [file Supplementary_file_2.docx]

**Supplemental file 2**

The purpose of these instructions is to ensure that instructors are prepared to teach a robust standardized pulmonary-based Qigong for patients with COPD.

**The Action Details of Pulmonary-based Qigong Exercise**

**Action 1: Rise-up position**

Bending elbows, supinating forearms with ten fingertips opposing; Then lifting palms slowly to the chest level, and inhaling through the nose. Pronating forearms and pressing palms down slowly to the navel; Bending knees to squat and turning palms outward, then moving forward slowly until arms are rounded, pursed-lips exhaling. Turning palms inward, stand up, and slowly retracting arms to the navel, inhaling through the nose. Crossing palms and hold each other for a while, eyes closed slightly, breathe naturally.

**Action 2: “Hu” sounding**

Bending knees slightly, opening hands until arms are rounded, and exhaling with pronouncing “HU”. Then stand up, taking back hands, inhaling through the nose. Take a break then repeat the exercise 6 times.

**Action 3: “Si” sounding**

Continues with previous movement. Straightening knees slowly, dropping palms naturally with palms facing upwards, ten fingertips opposing. Then, raising palms slowly to the chest level, and inhaling through the nose. Dropping elbows and facing the two palms, extending shoulder and expanding chest, heads up and neck down, exhaling slowly and inhaling through the nose (one breath cycle ). Bending knees slightly, pursed-lips exhaling and pronouncing “SI”, simultaneously loosing shoulders and extending necks, pushing hands forward slowly with the palms facing forward. Then, rotating wrists to turn palms facing inward, retracting hands to about 10 cm in front of chest, straightening knees slowly, and inhaling through the nose. Take a break then repeat the exercise 6 times.

**Action 4: Pushing up the sky to regulate the triple warmer**

Continues with previous movement. Dropping palms naturally with palms facing upwards, ten fingertips opposing. Crossing hands in front of the abdomen with palms facing upwards, breathe naturally. Raising up hands to the level of chest and inhaling through the nose. Then turning the forearms to make palms facing upwards, and gazing at hands. Raising up hands until the elbows straight, looking ahead ( two breath cycles ). Bending knees, dropping arms on both sides of the body, pursed-lips exhaling. Take a break then repeat the exercise 6 times.

**Action 5: Drawing a bow to shoot a vulture**

Continues with previous movement. Shifting center of gravity right, stepping left of left foot. Crossing hand in front of chest with the left hand outside, looking ahead, inhaling through the nose. Extending left arm to the left with “Ba”(八) palm, gazing at the left fingertips, flexing right arm with the right fist in front of right chest, and bending knees to squat, pursed-lips exhaling. Shifting center of gravity right, extending right arm to the right, inhaling through the nose, dropping arms on both sides of the body, retrieving left foot. The right action is opposite to the left but similar. One left and one right as 1 time, repeat the exercises 3 times.

**Action 6: The crane extension in the crane exercise**

Continues with previous movement. Dropping palms naturally with palms facing upwards, ten fingertips opposing; separating feet according to the shoulder-width. Crossing hand in front of abdomen, raising the crossed hands slowly up to head along the centerline, at the same time, shrug, neck down, and tail up, inhaling through the nose. Dropping the crossed hands slowly along the centerline, scattering hands in front of the abdomen and stretching arms back to the sides of body, stretching left lower limb back to the sides of body with straight right lower limb, pursed-lips exhaling.

Crossing hand in front of abdomen, raising the crossed hands slowly up to head along the center-line, dropping the crossed hands slowly along the center-line, scattering hands in front of the abdomen and stretching arms back to the sides of body, stretching right lower limb back to the sides of body with straight left lower limb. One left and one right as 1 time, repeat the exercises 3 times.

**Action 7: The crane fly in the crane exercise**

Continues with previous movement. Holding hands in front of abdomen with palms facing upwards. Abducting arms on both side of body to the shoulder level, raising left knee to the horizontal level with right lower limb straight, inhaling through the nose. Dropping arms on both side of body, dropping left knee, pursed-lips exhaling. Abducting arms on both side of body up to head, raising left knee to the horizontal level with right lower limb straight, inhaling through the nose. Dropping arms on both side of body, dropping left knee, pursed-lips exhaling. (when dropping arms and left knee, the supporting lower limb bending slightly; after the left toe touches the ground, raising left knee). One left and one right as 1 time, repeat the exercises 3 times.

**Action 8: Cross-armed iron staff**

Continues with previous movement. Holding hands in front of abdomen with palms facing upwards. Raising and then crossing arms in front of chest, abducting arms on both sides of body with palms facing upwards, inhaling through the nose. Turning palms to face downwards, standing with toes, looking forward and exhaling slowly. Maintain this posture 10 to 20 seconds, breathe naturally. Dropping palms in front of body with palms facing upwards, dropping heels on the ground.

**Action 9: Restore position**

Stand naturally, and the feet separated according to the shoulder-width. Raising arms on both sides of body while inhaling. Dropping arms in front of body while exhaling. Coordinate movements with minds. Then, crossing hands in front of abdomen, pressing and rubbing the abdomen 6 times. Closing eyes and regulating breath.

**Intervention Protocol of Pulmonary-based Qigong Exercise Group**

| Phase one | Sessions | Pulmonary-based Qigong (PQ) Exercise |
| --- | --- | --- |
|  | 1 | - 20min - introduction of PQ, including the history, theory and basic working principle. - 10min - warm-up - 15min - abdominal breathing and pursed-lips breathing - 10min - weight shift in standing position - 5min - calm-down |
|  | 2 | - 10min - warm-up - 15min - abdominal breathing and pursed-lips breathing - 10min - review - 20min - horse stance, “rise-up position” - 5min - calm-down |
|  | 3 | - 10min - warm-up - 10min - abdominal breathing and pursed-lips breathing - 10min - review - 20min - “‘hu’ sounding”, “‘si’sounding” - 10min - calm-down |
|  | 4 | - 10min - warm-up - 10min - abdominal breathing and pursed-lips breathing - 10min - review - 20min - “pushing up the sky to regulate the triple warmer”, “drawing a bow to shoot a vulture” - 10min - calm-down |
|  | 5 | - 10min - warm-up - 10min - abdominal breathing and pursed-lips breathing - 10min - review - 20min - “the crane extension in the crane exercise”, “the crane fly in the crane exercise” - 10min - calm-down |
|  | 6 | - 10min - warm-up - 10min - abdominal breathing and pursed-lips breathing - 10min - review - 20min - “cross-armed iron staff”, “restore position” - 10min - calm-down |
| Phase two | 7-24 | - 5min - warm-up - 5min - abdominal breathing and pursed-lips breathing - 40min - PQ exercise - 10min - calm-down |
